# Interferon signaling pathways in health and disease

**DOI:** 10.1186/s43556-025-00381-5

**Published:** 2025-12-08

**Authors:** Chunli Wen, Qingzhan Lan, Yunshan Wang, Yang Ni, Alice S. T. Wong, Duanrui Liu

**Affiliations:** 1 Department of Clinical Laboratory, Shandong Provincial Hospital, Shandong University, Jinan, Shandong 250021 People’s Republic of China; 2 Key Laboratory for Experimental Teratology of the Ministry of Education, Department of Cell Biology, School of Basic Medical Sciences, Cheeloo College of Medicine, Shandong University, Jinan, Shandong 250012 China; 3 Department of Clinical Laboratory, Shandong Provincial Hospital, Shandong First Medical University, Jinan, Shandong 250021 People’s Republic of China; 4 Institute of Oncology, Shandong Provincial Hospital, Shandong First Medical University, Jinan, Shandong 250021 People’s Republic of China; 5 School of Biological Sciences, The University of Hong Kong, Pokfulam, Hong Kong, SAR 999077 China

**Keywords:** Interferon signaling pathways, Autoimmune disease, Immune responses, Targeted therapeutic strategies

## Abstract

Interferons (IFNs) are a family of cytokines that orchestrate a wide range of antiviral, immunoregulatory, and antitumor activities. This review provides a comprehensive overview of the molecular mechanisms underlying IFN signaling, including both canonical JAK (janus kinases)-STAT (signal transducers and activators of transcription) pathways and non-canonical branches such as MAPK (mitogen-activated protein kinase) and PI3K (phosphoinositide 3-kinase)-AKT (protein kinase B)-mTOR (mechanistic target of rapamycin). The intricate interplay between these signaling modules and transcriptional, epigenetic, and post-transcriptional regulators is essential for maintaining immune homeostasis and tailoring context-dependent immune responses. Under physiological conditions, IFNs are essential for host defense, driving antiviral gene expression, activating innate immune cells, and shaping adaptive immune responses, including T and B cells. Conversely, dysregulation of IFN signaling contributes to the development of autoimmune diseases, neuroinflammation, cardiovascular disorders, and cancer. Tumor cells can exploit IFN-induced suppressive molecules to evade immune attack. The currently emerging therapeutic strategies of IFN signaling have evolved into a dual strategy: replacement therapy in immunodeficient states, and pathway inhibition in autoimmune conditions. Additionally, IFN-based combination therapies with immune checkpoint blockade and radiotherapy have demonstrated synergistic potential but require precise control of dosing and timing to avoid immune exhaustion. Advances in single-cell transcriptomics, proteomics, and metabolomics are providing novel insights into IFN heterogeneity, enabling the development of personalized IFN-based treatments. This review highlights the clinical implications and emerging strategies to harness or restrain IFN signaling for therapeutic benefit.

## Introduction

Interferons (IFNs) are a multifunctional family of cytokines that have profoundly shaped contemporary immunology since their discovery by Isaacs and Lindenmann in 1957 [1, 2]. Originally characterized for their potent antiviral activity, IFNs are now recognized as master regulators of immune homeostasis, exhibiting broad immunomodulatory, antiproliferative, and differentiation-inducing properties that extend far beyond antiviral defense [3, 4]. All IFNs are secreted ligands of specific cell surface receptors, activating intracellular signaling cascades that culminate in the transcriptional regulation of interferon-stimulated genes (ISGs) [5]. These ISGs encode a wide array of effector proteins that collectively restrict viral replication, modulate immune cell activity, and regulate processes as diverse as antigen presentation, cell-cycle arrest, and programmed cell death.

The canonical JAK-STAT signaling pathway has long been established as the principal mediator of IFN responses [6]. Importantly, emerging evidence has revealed that IFN signaling is far more complex, incorporating non-canonical pathways such as MAPK, PI3K-AKT-mTOR, and MEK (mitogen-activated protein kinase kinase) -ERK (extracellular signal-regulated kinase), along with interactions with epigenetic regulators (e.g., Setdb1) and post-translational modifiers (e.g., ISG15, IFITs) [7–10]. These regulatory layers enable dynamic modulation of IFN output, allowing cells to tailor immune responses to specific infectious, inflammatory, or carcinogenic signals.

Despite decades of investigation, the complexity of IFN signaling continues to unfold. A properly tuned interferon response serves as a guardian of life, whereas its dysregulation, whether excessive or insufficient, can become a disruptor of health [11]. Under physiological conditions, IFNs are constitutively expressed at low levels, supporting tissue homeostasis and priming the immune system against infections [12]. Upon viral invasion, IFNs rapidly induce an antiviral state within cells, directly inhibiting viral replication while activating natural killer (NK) cells and T cells to eliminate infected targets during the early stages of viral invasion. Beyond their direct antiviral effects, IFNs play a crucial role in modulating adaptive immunity. IFN signaling promotes T cell proliferation, survival, and effector differentiation in conjunction with T cell receptor (TCR) engagement [13]. Furthermore, IFNs enhance the capacity of dendritic cells (DCs) to produce B cell-stimulatory cytokines such as BAFF (B cell-activating factor) and APRIL (a proliferation-inducing ligand), thereby facilitating immunoglobulin class-switch recombination [9]. In addition, IFNs can directly target tumor cells by blocking cell-cycle progression and inducing apoptosis, highlighting their multifaceted immunoregulatory and antitumor potentials.

The dysregulation of IFN signaling pathways is increasingly recognized as a hallmark of numerous pathological processes. Chronic or excessive activation of interferon signaling not only contributes to autoimmune and neurodegenerative diseases but also facilitates tumor immune evasion and resistance [14]. Moreover, chronic IFN stimulation can profoundly affect vascular and metabolic homeostasis. Prolonged interferon exposure suppresses endothelial nitric oxide synthase (NOS3) expression and nitric oxide (NO) production, leading to endothelial dysfunction and promoting the progression of atherosclerosis [15]. Thus, understanding the intricate regulation and context-dependent effects of IFN signaling remains crucial for developing targeted therapies.

Overall, we emphasize the complexity and pleiotropic nature of this class of immune mediators, IFNs, which exert fundamental roles in maintaining physiological homeostasis as well as in diverse pathological contexts, sometimes even eliciting opposing effects. Understanding the context-dependent regulation, feedback mechanisms, and crosstalk among IFN pathways is crucial for developing targeted interventions that harness their protective functions while minimizing deleterious effects. This review provides a comprehensive overview of interferon signaling networks, molecular mechanisms, and pathophysiological roles across infectious, autoimmune, oncogenic, and neurodegenerative diseases. Highlighting the diverse roles of distinct interferon subtypes in specific pathological contexts will pave the way for the development of novel therapeutic interventions and potentially bring transformative impacts on human health.

## Basic mechanism of interferon signaling pathways

### IFNs receptor complexes

**Fig. 1:**
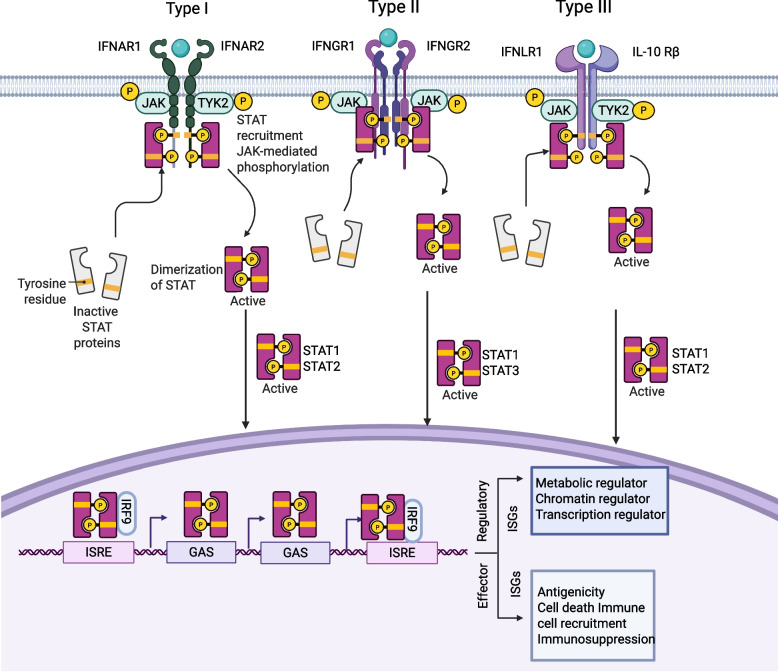
IFNs Receptor Complexes. Type I and Type III interferon receptors form heterodimers composed of the subunits IFNAR1 and IFNAR2, or IFNLR1 and IL10Rβ, respectively. Upon receptor dimerization, the associated kinases TYK2 and JAK1 are activated, leading to phosphorylation of STAT1 and STAT2. The phosphorylated STAT1-STAT2 heterodimer associates with IRF9 to form the transcription factor ISGF3, which binds to interferon-stimulated response elements (ISREs) and drives the expression of interferon-stimulated genes (ISGs). By contrast, Type II interferon first engages the IFNGR1 and IFNGR2 receptor complex, resulting in phosphorylation of STAT1 and STAT3. The phosphorylated STATs then form heterodimers that translocate into the nucleus and bind to gamma-activated sequence (GAS) elements, inducing ISG transcription. IFNAR1, interferon alpha/beta Receptor subunit 1; IFNAR2, interferon alpha/beta Receptor subunit 2; IFNGR1, interferon gamma receptor 1; IFNGR2, interferon gamma receptor 2; IFNLR1, interferon lambda receptor 1; IL10Rβ, interleukin-10 receptor beta; JAK1, janus kinase 1; TYK2, tyrosine kinase 2; STAT, signal transducer and activator of transcription 1; IRF9, interferon regulatory factor 9; ISGF3, interferon-stimulated gene factor 3. Figure created with BioRender.com

Although all IFNs share the capacity to induce antiviral states and coordinate immune activation, they are broadly categorized into three major classes, including type I, II, and III, based on sequence homology, receptor specificity, and evolutionary relationships. Each type activates distinct yet interconnected signaling cascades that collectively shape the immune landscape and maintain host defense (Table 1) [19, 20]. Type I interferon (IFN-I) primarily comprises multiple subtypes, including IFN-α, IFN-β, IFN-ε, IFN-κ, and IFN-ω, among which IFN-α and IFN-β are the most well-characterized and extensively studied [21]. IFN-α/β are produced by a broad spectrum of cell types, including monocytes, macrophages, B cells, T cells, platelets, epithelial cells, endothelial cells, and tumor cells, reflecting their central role in host antiviral defense and immune regulation [22]. Type I interferon receptors, encoded on human chromosome 21, comprise the subunits IFNAR1 (interferon α/β receptor subunit 1) and IFNAR2 (interferon α/β receptor subunit 2), both belonging to the class I cytokine receptor family [23]. Ligand binding activates the heterodimeric IFNAR1/IFNAR2 complex, which recruits and phosphorylates JAK and tyrosine kinase 2 (TYK2) (Fig. 1) [24, 25]. This phosphorylation cascade initiates the recruitment and activation of STAT proteins, particularly STAT1 and STAT2, which can heterodimerize and recruit IFN-regulatory factor 9 (IRF9) to form STAT1-STAT2-IRF9 tri-complex (ISGF3) [26, 27]. Subsequently, ISGF3 translocates into the nucleus and binds to interferon-stimulated response elements (ISREs) within the promoter regions of target genes, thereby initiating the transcription of a wide array of interferon-stimulated genes [26, 27].

**Table 1:** Characteristics of interferon receptor complexes

IFN type	Subtypes	Receptor complex	DNA binding motif	Activated kinases	Receptor distribution	References
Type I	IFN-α, IFN-β, IFN-ε, IFN-κ, IFN-ω	IFNAR1/IFNAR2	ISRE	JAK1/TYK2	Ubiquitously express	[16]
Type II	IFN-γ	IFNGR1/IFNGR2	GAS	JAK1/JAK2	Ubiquitously express	[17]
Type III	IFN-λ1, IFN-λ2, IFN-λ3, IFN-λ4	IFNLR1/IL-10Rβ	ISRE	JAK1/TYK2	Express on epithelial cells	[18]

*ISRE* interferon-stimulated response element, *GAS* gamma-activated sequence

Type II interferon (IFN-II) is represented solely by IFN-γ, which is primarily produced by activated immune cells, including T lymphocytes, B lymphocytes, natural killer (NK) cells, and natural killer T (NKT) cells, playing a pivotal role in both innate and adaptive immunity [28, 29]. Its expression is induced by cytokines such as IL-12 and IL-18 or through pattern recognition receptor (PRR) activation during infection or tissue damage [30]. The biological effects of IFN-γ are mediated through its specific receptor complex, interferon-gamma receptor (IFNGR), which consists of IFNGR1 (α chain) and IFNGR2 (β chain). Ligand-driven receptor dimerization is tightly controlled. Whereas IFNGR1 is constitutively expressed on all nucleated cells, IFNGR2 expression is subject to stringent regulation [31]. Upon ligand binding, the heterodimeric IFNGR complex activates Jak1 and Jak2 tyrosine kinases that bind to the intracellular domains of IFN-γR1 and IFN-γR2, respectively, leading to STAT1 and STAT3 phosphorylation [32, 33]. Translocation of STAT1 and STAT3 into the nucleus and bind to gamma-activated sequence (GAS) elements in the promoters of IFN-γ-stimulated genes, such as interferon regulatory factor 1 (IRF1), modulating the expression of genes involved in the secondary response to IFN-γ [29, 34]. Functionally, IFN-γ enhances macrophage activation, antigen presentation, and cytotoxicity, contributing to host defense and immune regulation [35].

Type III interferon (IFN-III), identified in 2003, comprise four members: IFN-λ1 (IL-29), IFN-λ2 (IL-28A), IFN-λ3 (IL-28B), and IFN-λ4 [36–38]. Type III interferon deliver signals through a heterodimeric receptor composed of IFNLR1 and IL-10Rβ, whose expression is largely restricted to epithelial tissues of the respiratory and gastrointestinal tracts [13]. This restricted distribution ensures localized antiviral protection while minimizing systemic inflammation [39, 40]. Produced mainly by epithelial cells and plasmacytoid dendritic cells, type III interferon provides first-line antiviral defense at mucosal barriers [41, 42]. Their expression is induced by viral recognition through RIG-I-like receptors (RLRs) and mitochondrial antiviral signaling protein (MAVS), which activate nuclear factor κB (NF-κB) and interferon regulatory factors (IRFs) [43, 44]. Type III interferon activates the JAK-STAT pathway upon receptor engagement, driving the transcription of antiviral ISGs [45].

Collectively, type I, II, and III interferons exhibit both shared and distinct molecular and cellular characteristics. A deeper understanding of these aspects will enable more precise modulation of IFN signaling outcomes in specific cell types and organ systems.

### JAK-STAT signal transduction pathway

IFNs regulate immune cell development and function through diverse signaling pathways, thereby influencing antimicrobial defense, autoimmunity, and cancer, as well as maintaining overall physiological homeostasis. The JAK-STAT signaling cascade is a tightly coordinated and rigorously regulated pathway central to IFN signal transduction. Ligand binding to IFN receptors induces activate the kinase domains (JH1) of the associated Janus kinase (JAK), leading to autophosphorylation on specific tyrosine residues. Activated JAK subsequently phosphorylates defined tyrosine sites within the intracellular domains of the receptor subunits (e.g., Tyr466 on IFNAR2, Tyr440 on IFNGR1), generating phosphotyrosine (pTyr) motifs [46]. These motifs serve as docking sites for STAT proteins, which engage via their Src homology 2 (SH2) domains and are recruited into proximity with the JAK complex [47, 48]. The STAT proteins are phosphorylated by activated JAK at their C-terminal tyrosine residues once recruited. This tyrosine phosphorylation drives the dimerization of STAT, leading to the formation of either homodimers or heterodimers. For instance, type I interferon induces the formation of STAT1-STAT2 heterodimers, while type II interferon promotes the assembly of STAT1-STAT3 homodimers. The formed STAT dimers harbor nuclear localization signals (NLSs) and translocate into the nucleus through nuclear pore complexes, a process that depends on the assistance of nuclear transport proteins (importin-α/β) [49, 50]. Upon DNA binding, STAT complexes recruit transcriptional coactivators such as CBP/p300 through their transactivation domains (TADs), initiating ISGs transcription [51]. The resulting ISGs products (e.g., MX1, OAS1, PKR) mediate diverse biological outcomes, ranging from antiviral defense and immune regulation to the control of cell proliferation and apoptosis.

The JAK-STAT pathway is subject to stringent negative regulation that fine-tunes both the amplitude and duration of signaling, thereby preventing aberrant activation that could trigger immunopathology or cellular dysfunction. Among the best characterized regulators are suppressor of cytokine signaling (SOCS) proteins, which suppress JAK kinase activity by binding to the JH1 catalytic domain and, through their SOCS box, recruit E3 ubiquitin ligase complexes to target JAK or STAT for proteasomal degradation [52, 53]. Protein inhibitors of activated STAT (PIAS) proteins, including PIAS1 and PIAS3, restrain STAT activity by blocking DNA binding of activated dimers or by promoting SUMOylation that attenuates their transcriptional potential [54, 55]. In parallel, tyrosine phosphatases such as SHP1 and SHP2 terminate signaling by dephosphorylating JAK or STAT at critical residues [56]. Together, these multilayered mechanisms ensure the spatial and temporal precision of JAK-STAT signaling, consolidating its role as a central hub of interferon responses and highlighting its therapeutic relevance as a target for clinical intervention.

### Non-canonical pathways

#### MAPK signaling pathway

**Fig. 2:**
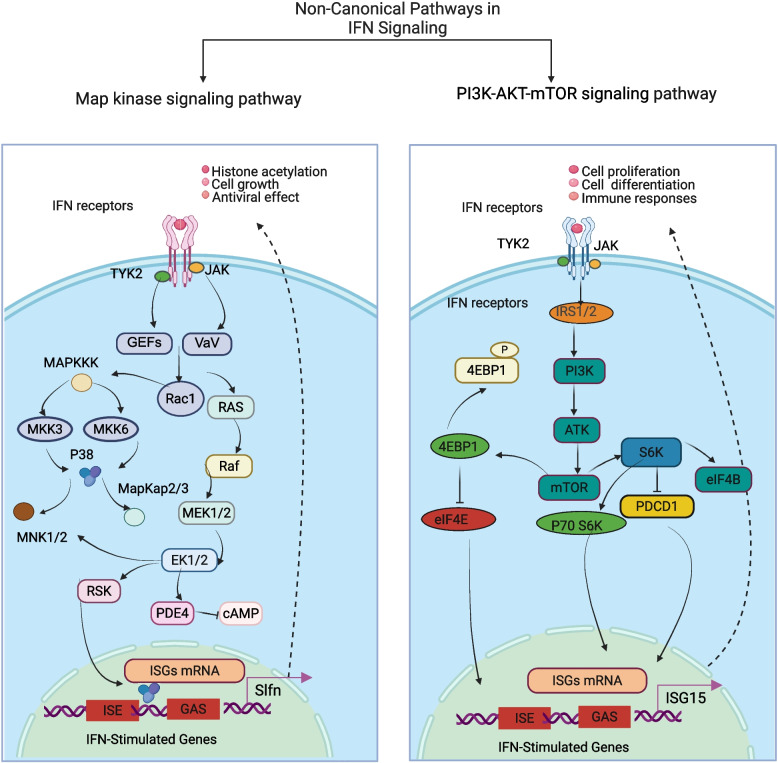
Non-Canonical Signaling Pathways in IFN signaling. Upon IFN receptor engagement, JAK and TYK2 kinases recruit guanine nucleotide exchange factors (GEFs) such as Vav, leading to the activation of Rac1 and Ras. These small GTPases initiate downstream phosphorylation cascades involving MAP kinase kinases 3 and 6 (MKK3/6) and MEK1/2, culminating in the activation of p38 MAPK and ERK1/2. Activated p38 and ERK regulate transcriptional programs through phosphorylation of downstream effectors (e.g., RSK, MNK1/2) and modulation of cAMP levels via PDE4. This pathway ultimately promotes the transcription of ISGs through ISRE and GAS elements, including genes of the Schlafen (Slfn) family, contributing to histone acetylation, cell growth regulation, and antiviral effects. Following IFN receptor activation, IRS1/2 recruits PI3K, leading to AKT activation. AKT subsequently stimulates mTOR signaling, promoting phosphorylation of downstream effectors such as S6K1, eIF4B, and PDCD4. Concurrently, phosphorylation of the translational repressor 4E-BP1 releases eIF4E, enabling cap-dependent mRNA translation. Collectively, these events enhance ISGs transcription and translation (e.g., ISG15), regulating cellular processes including proliferation, differentiation, metabolism, and immune responses. MAPK, mitogen-activated protein kinase; RSK, ribosomal S6 Kinase; MNK1/2, MAP Kinase Interacting serine/threonine-kinase 1/2; PDE4, Phosphodiesterase 4; IRS1/2, insulin receptor substrate 1/2;S6K1, ribosomal protein S6 kinase 1; eIF4B, eukaryotic initiation factor 4B; 4E-BP1, eukaryotic initiation factor 4E-binding protein 1; eIF4E, eukaryotic initiation factor 4E; PDCD4, programmed cell death 4. Figure created with BioRender.com

Beyond the canonical JAK-STAT cascade, IFN also initiates non-canonical signaling pathways that fine-tune transcriptional outcomes and broaden cellular responses (Fig. 2) [57, 58]. One such branch is the mitogen-activated protein kinase (MAPK) pathway. In this pathway, JAK1 phosphorylates the guanine nucleotide exchange factor Vav, which activates the small GTPase Rac1(Ras-related C3 botulinum toxin substrate 1), a process specifically achieved by facilitating the conversion of Rac1 from its inactive GDP-bound state to the active GTP-bound state [59]. Active Rac1 subsequently engages downstream effectors to specifically activate MAP kinase kinases 3 and 6 (MAPKK3/6, also known as MKK3/6) [60]. As intermediate kinases within the MAPK cascade, phosphorylated MKK3/6 further activate p38 MAPK by targeting its critical threonine/tyrosine residues (Thr180/Tyr182), converting it into its active form. Once translocated into the nucleus, activated p38 MAPK phosphorylates transcription factors such as ATF2 and NF-κB or co-activators, thereby enhancing their binding to ISRE elements within ISG promoters (e.g., the Schlafen gene family) or to GAS elements, ultimately promoting ISG transcription. This signaling branch modulates inflammation, stress tolerance, and apoptosis, and cooperates with interferon-mediated antiviral defense by inhibiting viral replication and promoting immune cell activation.

In contrast to the broad regulatory roles of the p38 MAPK pathway, the MEK-ERK branch exhibits subtype-specific functions across different IFNs. In type I interferon signaling, MEK-ERK activation directly phosphorylates phosphodiesterase 4 (PDE4), enhancing its enzymatic activity. As PDE4 degrades cyclic AMP (cAMP), its activation markedly reduces intracellular cAMP levels in regulatory T (Treg) cells. Lower cAMP weakens Treg-mediated immunosuppression, releasing effector T cells (e.g., Th1 and cytotoxic T lymphocytes) from inhibition and indirectly amplifying IFN-driven anti-tumor and antiviral immunity [61, 62]. In type II interferon signaling, the MEK-ERK branch functions not as an independent effector but as an enhancer of the canonical JAK-STAT pathway. Activated ERK phosphorylates STAT1 at specific serine residues (e.g., Ser727), increasing the DNA-binding affinity of STAT1 homodimers for GAS elements. This modification strengthens STAT1-dependent ISG transcription, such as IFN-γ-inducible chemokine CXCL10, thereby augmenting the pro-inflammatory and antitumor activities of IFN-γ [63]. For type III interferon, whose receptors are predominantly expressed on epithelial cells of the respiratory and intestinal tracts, MEK-ERK signaling is primarily linked to epithelial growth regulation. Activated MEK-ERK phosphorylates ribosomal S6 kinase 1 (RSK1), which in turn modulates the expression and activity of the cell cycle inhibitor p21/WAF1/CIP1. This regulation leads to G1/S cell cycle arrest in epithelial cells, effectively limiting their proliferation [64]. Such growth arrest is a critical component of IFN-λ-mediated epithelial antiviral defense, restricting viral dissemination by suppressing the proliferation of infected epithelial cells. Taken together, MAPK signaling provides a complementary layer of regulation by phosphorylating transcriptional co-factors, modulating cell cycle regulators, and fine-tuning immune cell activity. This auxiliary precision underscores the cooperative nature of MAPK branches in shaping the breadth and specificity of IFN responses.

#### PI3K-AKT-mTOR signaling pathway

A second non-canonical branch involves the PI3K-AKT-mTOR signaling axis, which is critical for cell survival, metabolism, and immune regulation during IFN responses [65]. Type I interferon rapidly activates this pathway, resulting in phosphorylation of p70 S6 kinase (S6K1) and its effector, the S6 ribosomal protein [66]. In parallel, type I interferon signaling phosphorylates the translational repressor 4E-BP1, releasing it from eukaryotic initiation factor 4E (eIF4E) and thereby enabling cap-dependent translation [67]. In addition, the IFN-activated form of S6K can phosphorylate serine 67 (Ser67) of the tumor suppressor protein, programmed cell death 4 (PDCD4), which leads to the interaction between PDCD4 and the ubiquitin ligase β-transducin repeat-containing protein (β-TRCP), causing the degradation of PDCD4 [68]. PDCD4 degradation ultimately facilitates expression of several ISG protein products that play critical roles in the generation of IFN responses, including IFN-stimulated gene 15 (ISG15), p21WAF1/CIP1, and Schlafen 5 (SLFN5). In summary, activation of the PI3K-AKT-mTOR signaling branch complements the canonical JAK-STAT cascade, fine-tuning interferon-mediated responses by coupling antiviral signaling with translational control, metabolic adaptation, and cell survival.

### Functions of downstream effectors

Once activation of these signaling pathways, interferons induce the expression of downstream effector molecules known as ISGs. Among them, the interferon-induced proteins with tetratricopeptide repeats (IFITs) constitute one of the most robustly upregulated ISG families in response to interferon stimulation [69, 70]. In humans, the IFITs gene cluster is located on chromosome 10 and comprises at least four members:IFIT1/ISG56, IFIT2/ISG54, IFIT3/ISG60, and IFIT5/ISG58 [71]. Proteins of the IFITs family are characterized by the presence of tetratricopeptide repeat (TPR) domains, composed of tandem 34-amino acid motifs that fold into helix-turn-helix structures. These domains facilitate protein–protein interactions, enabling IFITs to function as scaffolding molecules or regulatory factors in diverse immune and antiviral pathways [69, 72].

Under physiological conditions, IFITs are expressed at low basal levels and are primarily localized in the cytoplasm. Their transcription is strongly induced by IFNs (particularly type I interferon and type II interferon), viral infections, and pathogen-associated molecular patterns (PAMPs) such as dsRNA and lipopolysaccharides [73]. Beyond antiviral defense, recent studies have demonstrated that the IFITs family also plays critical roles in cancer progression and metastasis, with aberrant expression reported in oral squamous cell carcinoma, osteosarcoma, pancreatic cancer, and bladder cancer [74]. IFITs proteins can remodel extracellular matrix (ECM) by decreasing the expression of epithelial markers such as E-cadherin and increasing the expression of interstitial markers such as N-cadherin, as well as releasing inhibitory cytokines, such as IL-1β and IL-6 that counteract T cell function and promote cancer cell migration, metastatic potential and proliferation [75]. In tumor contexts, chronic IFN signaling can sustain STAT1 activation, leading to persistent IFITs upregulation [76, 77]. This prolonged activation can promote the production of inflammatory cytokines, such as IL-6 and IL-4, and tumor metabolism-related factors, such as indoleamine-2,3-dioxygenase, facilitating the formation of the tumor microenvironment (TME) and attenuating the effects of immune checkpoint blockade therapies, including PD-1 (programmed cell death protein 1)/PD-L1 (programmed cell death ligand 1) inhibitors. As downstream effectors of interferons, ISGs, particularly the IFITs family, not only mediate broad antiviral activities but also exert context-dependent roles in tumor progression and immune modulation. While acute IFN responses promote pathogen clearance and antitumor immunity, chronic or dysregulated signaling may sustain STAT1-driven IFITs upregulation, fostering immunosuppressive reprogramming and therapeutic resistance.

In summary, IFN signaling integrates canonical JAK-STAT cascades with non-canonical branches such as MAPK and PI3K-mTOR, establishing a finely tuned equilibrium among antiviral defense, immune regulation, and tissue homeostasis. These sophisticated feedback loops tightly control both the magnitude and duration of interferon production and signaling activity. Consequently, a deeper understanding of of these interconnected pathways is essential for selectively enhancing or suppressing specific interferon signaling pathways to intervene in diverse pathological processes.

## IFN signaling in physiological health

### Antiviral defense mechanisms

#### Key steps in blocking viral replication

**Fig. 3:**
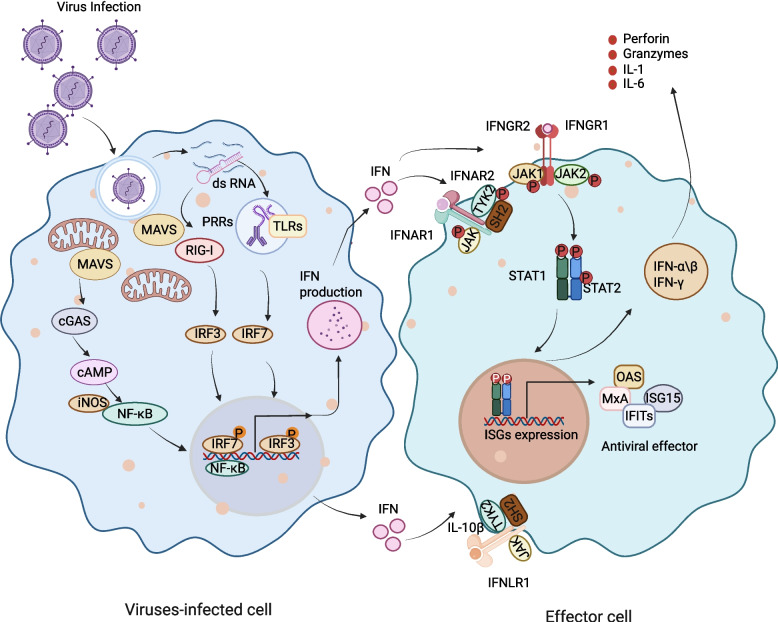
Schematic overview of interferon signaling in antiviral defense. Viral infection triggers recognition of pathogen-associated molecular patterns (PAMPs), such as double-stranded RNA, by pattern recognition receptors (PRRs) including RIG-I, Toll-like receptors (TLRs)and cytoplasmic cyclic GMP-AMP synthase. Downstream activation of MAVS, cGAS, NF-κB, IRF3, and IRF7 induces IFN production. Secreted IFNs bind to cognate receptors (IFNAR1/2, IFNLR1, or IFNGR1/2) on effector cells, activating JAK1, TYK2, and JAK2 kinases, which phosphorylate STAT1/STAT2. These transcription factors drive the expression of ISGs, including ISG15, IFITs, OAS, and MxA, which mediate antiviral functions through inhibition of viral replication, degradation of viral RNA, and modulation of host immune responses. RIG-I, retinoic acid-inducible gene-I; MAVS, mitochondrial antiviral signaling protein; cGAS, cyclic GMP-AMP synthase; OAS, 2‘−5'-Oligoadenylate Synthase; MxA, myxovirus resistance protein A. Figure created with BioRender.com

IFNs execute diverse functions spanning antiviral defense, amplification of innate immune responses, and preservation of immune balance through cell type-specific molecular programs. First characterized for their extraordinary ability to induce cellular resistance to viral infection, interferons signal through ubiquitously expressed receptors to activate extensive antiviral gene networks (Fig. 3) [78]. The antiviral activity of interferons is initiated when pathogen-associated molecular patterns (PAMPs) are sensed by host pattern recognition receptors (PRRs) [79]. The PPRs of host cells mainly include Toll-like receptors (TLRs), RIG-I-like receptors (RLRs), and cytoplasmic cyclic GMP-AMP synthase [80]. PAMP-PRR interactions activate interferon regulatory factors (IRF3, IRF7), which bind to IFN gene promoters and initiate transcription [81]. Secreted IFNs then engage their cognate receptors and activate the JAK-STAT pathway via autocrine or paracrine signaling, leading to robust induction of ISGs [82]. ISGs restrict viral replication through degrading nucleic acids and inhibiting gene expression, protein synthesis, and viral particle assembly [5]. For instance, ISG15, a ubiquitin-like modifier, conjugates with viral and host proteins to alter their stability and localization [83]. The myxovirus resistance (Mx) genes represent some of the most extensively studied ISGs with established roles in antiviral defense [84, 85]. Mx proteins exist in two major isoforms, Mx1 and Mx2, which are prototypical antiviral mediators that disrupt viral replication by preventing nucleocapsid trafficking and sequestering viral components. Beyond the Mx family, the OAS (2′−5′-Oligoadenylate Synthetases) and protein kinase R (PKR) are two additional families of ISGs that mediate antiviral activity by inhibiting protein synthesis. OAS enzymes catalyze the generation of 2′−5′-linked oligoadenylates from ATP, which can contribute to activate latent RNase L and degrade viral RNA. Similarly, PKR exert antiviral effects in cells exposed to double-stranded RNA by blocking protein translation [86]. PKR phosphorylates the eukaryotic initiation factor eIF2 upon activation by type I and type III interferons, sequestering eIF2B and blocking the GDP-to-GTP exchange required for viral RNA synthesis [87]. Mechanistically, IFNs primarily regulate the transcription of numerous ISGs through the JAK-STAT signaling pathway. Acting as potent effectors of cell-autonomous immunity, ISGs disrupt viral replication at multiple checkpoints, from cellular entry to virion assembly and release. This multifaceted antiviral program endows host cells with broad resistance against RNA and DNA viruses, as well as intracellular bacteria and parasites, highlighting the evolutionary versatility of IFN-mediated immune protection.

#### Enhancement of innate immunity

Beyond directly inhibiting viral replication, IFNs govern a multilayered signaling network that activates and coordinates the innate immune system, achieving precise amplification and integration from pathogen recognition to immune effector execution [88]. As the foremost physical barrier against infection, epithelial surfaces rely heavily on type III interferon to maintain frontline innate immune protection [89, 90]. Through engagement of its receptor complex, type III interferon triggers a rapid and localized induction of ISGs within the respiratory and intestinal epithelia, orchestrating the clearance of infected cells and the restoration of tissue integrity [91]. Besides antiviral defense, type III interferon tempers inflammation by limiting neutrophil degranulation and reactive oxygen species production. When pathogens breach this mucosal line of defense, type I and type II interferons subsequently assume control, coordinating a systemic immune response to contain and eliminate infection. Type I interferon enhances antigen visibility and antigen-presenting cell (APC) function through upregulation of MHC complex [92, 93]. Simultaneously, they drive macrophage production of inducible nitric oxide synthase (iNOS), leading to nitric oxide (NO) production for pathogen killing [94]. Concurrently, type I interferon promotes the secretion of pro-inflammatory cytokines such as TNF-α, IL-1β, and IL-6, which recruits immune cells and activates the NF-κB and MAPK pathways, amplifying inflammatory and phagocytic responses [95]. Furthermore, IFN-γ critically modulates NK cells function by upregulating activating receptors, most notably natural killer group 2 member D (NKG2D) [96]. Engagement of NKG2D with its ligands expressed on the surface of virally infected cells triggers NK cell degranulation, leading to the release of cytotoxic mediators such as perforin and granzymes, which mediate the lysis and clearance of infected cells [97]. In all, IFNs act as pivotal regulators that bridge antiviral transcriptional programs with the effector functions of innate immune cells. This dual role ensures both direct viral suppression and coordinated immune cell recruitment, suggesting that fine-tuning IFN signaling may be a key therapeutic strategy for balancing antiviral defense with inflammation control.

### Regulation of adaptive immune responses

#### T cell activation and differentiation

**Fig. 4:**
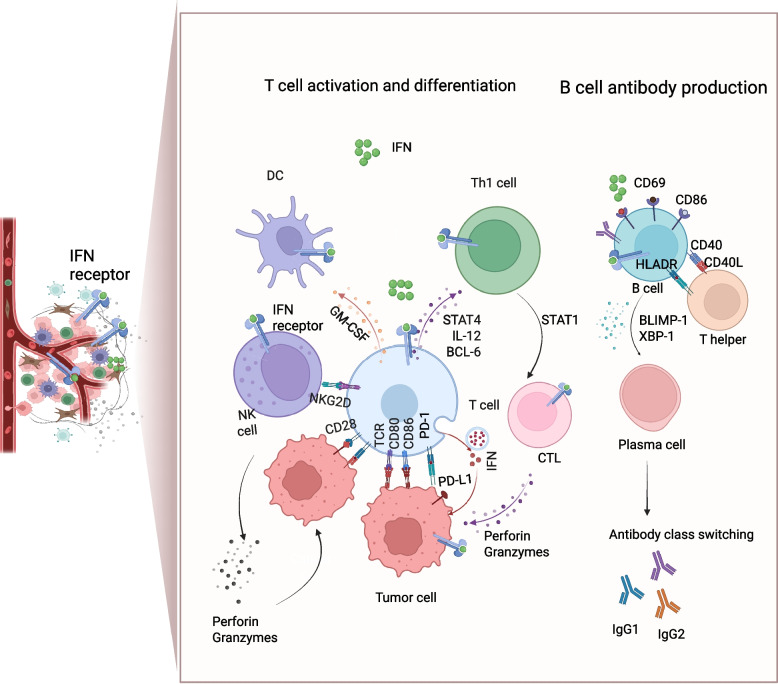
IFN-mediated regulation of T and B cell immunity. IFN signaling promotes dendritic cell (DC) maturation under GM-CSF and sustains MHC class II expression to enhance antigen presentation. Type I interferon activates NK cells through NKG2D, inducing cytotoxic responses via perforin and granzymes. In T cells, type I interferon drives STAT4 activation and upregulation of IL-12, BCL-6, CXCR5, and PD-1, favoring Th1 differentiation. IFN signaling further augments cytotoxic T lymphocyte (CTL) activity through STAT1, leading to enhanced perforin and granzyme release. IFN-induced upregulation of CD40, CD69, CD86, and MHC-II (HLA-DR) on B cells facilitates interaction with CD40L-expressing T helper cells, sustaining clonal expansion. Moreover, IFN promotes expression of transcription factors BLIMP-1 and XBP-1, supporting plasma cell differentiation and antibody secretion. Together, these processes drive antibody class switching, including IgG1 and IgG2 isotypes, tailoring humoral responses. GM-CSF, granulocyte–macrophage colony-stimulating factor; NKG2D, natural killer group 2, member D; BCL-6, B-cell lymphoma 6; CXCR5, C-X-C chemokine receptor type 5; PD-1, programmed cell death protein 1; BLIMP-1, B-lymphocyte-induced maturation protein-1; XBP-1, X-box binding protein 1. Figure created with BioRender.com

IFNs serve as a molecular bridge between innate and adaptive immunity. By influencing antigen presentation, cytokine signal transduction, and lymphocyte differentiation, they ensure that early pathogen sensing translates into precise and sustained adaptive immune responses. T cell activation requires three integrated signals: T cell receptor (TCR) recognition of peptide-MHC complexes, costimulatory receptor-ligand interactions (e.g., CD28/CD80/CD86), and cytokine signaling, which guides lineage fate (Fig. 4) [98]. Type I interferon (IFN-α/β) promotes monocyte differentiation into mature DCs under granulocyte–macrophage colony-stimulating factor (GM-CSF), enhancing cell surface expression of MHC molecules and co-stimulatory molecules, including CD80 and CD86, which is associated with increased ability to stimulate T cell [99–101]. Type I interferon augments CD8⁺ T cell cytotoxicity by activating the IFNAR pathway [98, 102]. IFN-γ favors Th1 differentiation via STAT4 activation, modulating genes such as interleukin-12 (IL-12), B cell lymphoma 6 (BCL-6), chemokine receptor CXCR5, and PD-1 [103, 104]. Following the activation and lineage commitment of T cells, interferons further refine their effector and memory potential through precise molecular regulation [105]. Exposure of CD8⁺ T cells to IFN-γ induces upregulation of the IL-2 receptor, the transcription factor T-bet, and granzyme expression. IL-2 responsiveness is essential for generating cytolytic CD8⁺ T cells, while granzyme directly mediates target cell lysis, underscoring the indispensable role of IFN-γ in licensing CD8⁺ T cell effector functions [106]. Type I interferon further sustains the effector capacity and tissue trafficking of memory T cells during secondary infections. Studies demonstrate that type I interferon drives the activation of circulating memory T cells, which exerts cytotoxicity and migrate to the lung tissues, promoting the production of key factors such as IL-15 and IL-18 by inflammatory monocytes to maintain the survival and function of memory CD8⁺ T cells following viral infection [107]. Furthermore, another study demonstrated that a DNA vaccine encoding both HIV antigens and IFN-λ elicited significantly stronger antigen-specific CD8⁺ T cell cytotoxic responses compared with a control vaccine encoding HIV antigens alone [108, 109]. Furthermore, IFN-λ can also induce a persistent Th1-biased immune response, providing long-term protection against viral and tumor infections [110, 111]. Collectively, IFNs shape robust antiviral and antitumor immunity by orchestrating T cell differentiation, proliferation, and effector functions.

#### B cell antibody production

Following their pivotal role in modulating T cell activation and differentiation, IFNs also exert profound regulatory effects on B cell biology, governing humoral immune responses through the control of antibody production, class switching, and affinity maturation [112]. Type I interferon upregulates surface molecules including CD40, CD69, CD86, and MHC-II, which promote T-B cell interactions and antigen presentation [113]. CD40 strengthens B cell-T cell crosstalk through its interaction with CD40L on T helper cells [114]. CD69 can form complexes with S1P1 to promote the retention of circulating B cells in lymphoid tissues, facilitating immune activation [115]. CD86, as a costimulatory receptor, enhances the interaction between B cells and T cells [116]. MHC class II can enhance antigen presentation and BCR-mediated antigen recognition, promoting subsequent B cell activation and clonal expansion.

In addition, IFNs promote B cell maturation into plasma cells capable of robust antibody secretion. Type I interferon can drive CD40-induced B cells toward plasmablasts lacking immediate immunoglobulin (Ig) secretion, which are subsequently converted into fully functional, Ig-secreting plasma cells by IL-6 [117]. In memory B cells, IFN-α induces transcription factors such as B lymphocyte-induced maturation protein 1 (BLIMP-1) and X-box binding protein 1 (XBP-1), supporting antibody secretion and class-switch recombination [118]. Moreover, type I interferon enhances T-bet expression, favoring IgG2a production [119, 120], while IFN-γ suppresses IgG1 but promotes IgG2, thereby skewing the humoral response toward a Th1 phenotype [121]. Collectively, IFNs precisely shape antibody isotypes to match specific pathogen classes by regulating B cell differentiation and antibody class switching.

Together, these findings underscore the multifaceted roles of interferons as master coordinators of adaptive immunity. By simultaneously regulating T cell polarization and B cell antibody responses, IFNs ensure that cellular and humoral immunity are functionally aligned to achieve effective pathogen clearance and long-term immune protection.

### Maintenance of immunological homeostasis

#### Suppressive effects on autoimmune diseases

The basal IFNs characteristics possess significant physiological functions, which help maintain the homeostasis of tissues and enable the body to be in a prepared state to resist infections [122]. In the acute experimental colitis model induced by dextran sulfate sodium (DSS), the deletion of the Ifnar1 would exacerbate the disease progression, whereas administration of recombinant type I interferon exerts protective effects against colitis through acting on myeloid cells [123]. Clinically, type I interferon, particularly IFN-β, has been successfully applied in the treatment of multiple sclerosis (MS). IFN-β was the first immunomodulatory drug authorized for MS therapy in 1993. Its mechanism of action involves downregulating the expression of MHC class II on antigen-presenting cells, inducing regulatory T cells and the IL-10, inhibiting T cell proliferation, as well as reducing the expression of adhesion molecules and suppressing the migration of T cells across the blood–brain barrier [124, 125]. IFN-γ can delay the onset and reduces the severity of seizures induced by the gamma-aminobutyric acid receptor (GABA) antagonist pentylenetetrazol [126, 127]. Additionally, studies in murine models demonstrate that IFN-λ1 suppresses joint inflammation in collagen-induced arthritis by inhibiting neutrophil recruitment [128]. In rheumatoid arthritis (RA), type III interferon can ameliorate joint inflammation by inhibiting the number of proinflammatory IL-17-producing Th17 and γδ T cells, as well as IL-1β-positive neutrophils in the joints and inguinal lymph nodes. The receptor for type III interferon (IFNLR1/IL-10R2) is mainly expressed on epithelial cells and immune cells (such as T cells and neutrophils), with a relatively restricted tissue distribution. This gives type III interferon a more local specificity in its anti-inflammatory effects, making it one of the research hotspots in autoimmune diseases like RA [129]. The type I interferon signaling pathway contributes to the homeostasis of Treg/Th17 cells in autoimmune hepatitis through the regulation of STAT3 and STAT5 [130]. STAT3 promotes Th17 differentiation by binding directly to the promoters of IL-17A and IL-17F and upregulating key transcription factors such as RORγt and RORα [131]. In contrast, STAT5 supports Treg stability by binding to the CNS2 region of the FOXP3 locus, preserving its heritable expression and protecting Tregs from inflammatory cytokine signals. Additionally, STAT5 acts as a negative regulator of Th17 differentiation by competing with STAT3 for shared binding sites on the IL-17A promoter and suppressing IL-17 production [132]. IFNs exert effects in autoimmune diseases through multi-dimensional immunomodulatory mechanisms, primarily by fine-tuning the balance between pro-inflammatory and regulatory immune cells. This includes inhibiting pathogenic T cell subsets (e.g., Th17) and neutrophil activation, while promoting regulatory circuits such as Treg development and anti-inflammatory cytokine production. Overall, by modulating the functions of specific tissue-resident and immune cells under diverse microenvironmental contexts, interferons exert protective effects that suppress disease progression. However, the heterogeneity of autoimmune diseases, including differences in clinical manifestations, therapeutic exposure, and genetic background, renders interferon activity multifaceted and context-dependent, with outcomes that may vary across disease states and patient populations.

#### Maintenance of inflammatory balance

Interferons act not only as initiators of immune defense but also as guardians of inflammatory equilibrium, ensuring that immune activation is proportionate and transient rather than chronic and pathogenic. IFNs reshape the cytokine microenvironment by driving the production of ISGs to prevent the overexpression of inflammatory factors, such as suppressors of cytokines signaling (SOCS) [133–137]. SOCS3 primarily binds to the GP130 subunit to inhibit the signal transduction of cytokines in the IL-6 family. This binding reduces the IL-6-induced activation of STAT3, which in turn suppresses the expression of downstream inflammation-related genes [53, 138]. Meanwhile, ISGs can also induce the production of anti-inflammatory cytokines like IL-10. IL-10 targets interferon regulatory factor (IRF) transcription factors through epigenetic mechanisms, inhibiting the expression of IFN and inflammation-responsive genes [139]. Additionally, IL-10 can suppress the expression of NF-κB target genes (e.g., TNF and IL-6) in monocytes, DCs, and macrophages, exerting its anti-inflammatory effects [140]. IFN-γ signaling in antigen-presenting cells (APCs) leads to the upregulated expression of costimulatory molecules and cytokines involved in generating effective T cell responses, such as intercellular adhesion molecule 1 (ICAM-1), CD86, IL-1β, and interleukin-12p70 (IL-12p70), facilitating the recruitment of immune effector cells to infection sites [141–143]. IFN-γ can promote the polarization of macrophages toward the M1 phenotype and produce pro-inflammatory cytokines including IL-1β, TNF-α, IL-12, IL-18, and IL-23 [144]. Additionally, IFN-γ can further facilitate its own production by enhancing the polarization of T helper 1 (Th1) cells and activating STAT4, ultimately forming a "IFN-γ-STAT4-Th1-IFN-γ" positive regulatory loop that modulates the expression of inflammatory factors [145]. In the early stage of bacterial infection, IL-1α and IL-1β limit the production of type I interferon by directly down-regulating transcription and mediating the production of PGE2 through PTGS2. Meanwhile, type I interferon weakens the IL-1α/β signal by inducing IL-10, IL-1R2, IL-1RA and CH25H, maintaining the balance of inflammatory factors [146]. IFNs act as key regulators of immune resolution through these pathways, ensuring that inflammation is effectively terminated once pathogens are cleared and that tissue homeostasis is restored. In summary, IFNs act as master regulators of inflammatory resolution, fine-tuning cytokine networks to ensure the controlled termination of immune responses once pathogens are cleared. Through reciprocal regulatory loops involving IL-10, SOCS proteins, and IRFs, IFNs maintain the delicate balance between immune activation and suppression.

#### Antitumor mechanisms of IFNs

The pleiotropic antitumor functions of IFNs have been widely recognized over the past few decades. IFNs orchestrate a comprehensive anti-tumor network through the synergistic integration of direct tumor cell-intrinsic effects and indirect immunomodulation of the TME. IFN-α induces apoptosis through multiple coordinated mechanisms. It enhances the expression of tumor necrosis factor-related apoptosis-inducing ligand (TRAIL) and its receptors DR4 and DR5, activating caspase-dependent apoptotic cascades. Concurrently, IFN-α upregulates Fas ligand (FasL) and TNF-α, which engage corresponding death receptors on tumor cells to trigger the extrinsic apoptotic pathway [134]. In addition, IFN-α downregulates the anti-apoptotic protein Bcl-2 while upregulating the pro-apoptotic protein Bax via activation of the STAT1/STAT6/TGF-β signaling axis, resulting in mitochondrial membrane permeabilization and activation of the intrinsic apoptotic pathway. IFN-ε exerts a direct antitumor effect on ovarian cancer cells by suppressing tumor growth and promoting tumor rejection responses [147]. IFN-γ was first reported to play a functional role in tumor immunity in 1994 [148]. A critical function of IFN-γ is the enhancement of tumor immunogenicity through enhancing antigen presentation and rendering cells more sensitive to immune surveillance [149]. Macrophages exposuring to IFN-γ can secrete IL-12 and upregulate MHC class II molecules, reinforcing Th1 polarization and amplifying CTL responses [150]. Th1 CD4⁺ T cells subsequently produce IL-2 and IFN-γ, which collectively support the differentiation of fully functional cytolytic CD8⁺ T cells capable of eliminating MHC class I-expressing tumor cells [151, 152]. Simultaneously, IFN-γ induces the expression of chemokine ligands such as CXCL9 and CXCL10, which bind to the CXCR3 receptor on effector T cells and NK cells, promoting their robust infiltration into the tumor microenvironment and effectively suppressing tumor growth [153]. In addition to directly inducing apoptosis in tumor cells, IFN-γ can also act on the tumor stroma and endothelial cells to enhance antitumor immunity. IFN-γ directly suppresses the expression of pro-angiogenic factors, including vascular endothelial growth factor (VEGF) and basic fibroblast growth factor (bFGF), while upregulating anti-angiogenic mediators such as thrombospondin-1, which can inhibit tumor neovascularization [154]. Moreover, IFN fine-tunes immune activation by modulating the expression of ubiquitin-specific peptidase 18 (USP18), a key negative regulator of IFN signaling, which can prevent excessive immune activation and exhaustion, sustaining tumor cell sensitivity to immune surveillance [155]. Type III interferon has also been implicated in antitumor immune responses [156]. Overexpression of type III interferon in mouse models has been shown to increase MHC-I expression, enhance tumor-infiltrating lymphocyte recruitment, and suppress tumor growth and metastasis [157]. However, recent evidence further indicates that the duration of IFN signaling is a critical determinant of immune outcome. Prolonged or excessive IFN stimulation can drive immune cell dysfunction or even promote tumor progression [158]. In particular, sustained upregulation of IFN-γ induces the expression of PD-L1, which binds to PD-1 on effector T cells and dampens antitumor immunity [159]. Collectively, these findings highlight the multifaceted and context-dependent roles of interferons in cancer, underscoring the need for deeper mechanistic understanding to harness their therapeutic potential effectively.

Together, interferon signaling, while traditionally recognized by its antiviral and immunostimulatory functions, also plays indispensable roles in maintaining physiological equilibrium under non-infectious conditions. In the steady state, IFN signaling ensures basal activation of key ISGs that sustain immune vigilance and protect against latent viral reactivation. The level of IFNs within tissues modulates shapes the composition and responsiveness of innate and adaptive immune cells. Elucidating the context-dependent mechanisms governing these processes is essential for developing targeted therapeutic strategies that harness interferon activity to promote health and combat disease.

## Dysregulated IFN signaling in disease

### Immune-related diseases

**Fig. 5:**
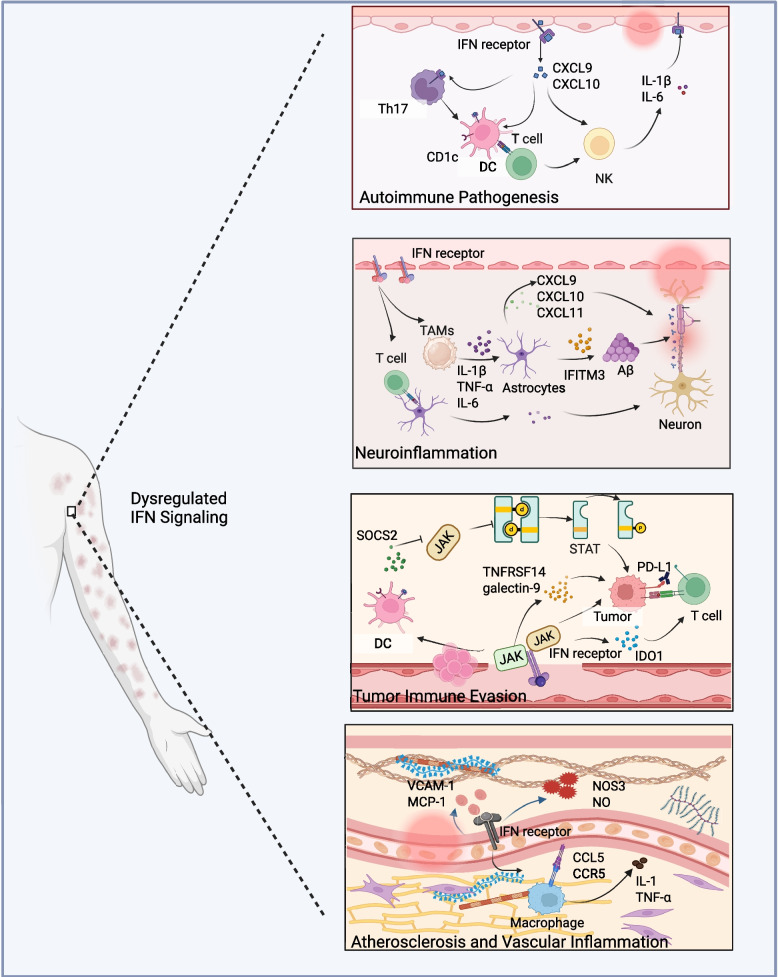
Pathological consequences of dysregulated IFN signaling. IFN-driven induction of CXCL9 and CXCL10 promotes DC-T cell-Th17 interactions and enhances IL-1β and IL-6 production, sustaining autoreactive responses and NK cell activation in autoimmune pathogenesis. IFN-stimulated astrocytes and microglia release IL-1β, TNF-α, and IL-6, while IFITM3 upregulation and chemokine secretion (CXCL9, CXCL10, CXCL11) exacerbate T cell infiltration and amyloid-β (Aβ)-associated neuronal injury in neuroinflammation. Chronic IFN signaling induces SOCS2, IDO1, and checkpoint pathways (PD-L1, galectin-9, TNFRSF14), dampening DC and T cell activation via JAK-STAT modulation and promoting tumor survival and evasion. Dysregulated IFN responses in endothelial cells and macrophages upregulate VCAM1, MCP-1, CCR5, and pro-inflammatory cytokines (IL-1, TNF-α), while NOS3-derived NO and CCL5-mediated recruitment perpetuate vascular injury and plaque progression in atherosclerosis and vascular inflammation. TNFRSF14, tumor necrosis factor receptor superfamily member 14; VCAM1, vascular cell Adhesion molecule 1; MCP-1, monocyte chemoattractant protein-1; CCR5, C–C chemokine receptor type 5. Figure created with BioRender.com

The relative balance of the activation and inhibition mechanisms induced by IFNs determines the overall functional outcome. An unrestricted response to pathogens or pathogen-associated molecular patterns can lead to persistent interferon signal transduction and trigger related diseases (Fig. 5) [160–162]. The dysregulated IFNs response play a pivotal role in both the initiation and perpetuation of systemic lupus erythematosus (SLE) [163]. Up to 80% of SLE patients exhibit a persistently hyperactivated type I interferon signature, characterized by elevated circulating interferon levels and enhanced expression of ISGs, which strongly correlate with disease severity, flare frequency, and therapeutic resistance [164, 165]. Mechanistically, aberrant activation of nucleic acid-sensing pathways, most notably Toll-like receptors TLR7 and TLR9, in conjunction with the cytosolic cGAS-STING axis, drives excessive type I interferon production in response to self-derived nucleic acids [24]. Multiple cellular sources, including monocytes, macrophages, follicular dendritic cells, and keratinocytes, have been identified as major contributors to this heightened type I interferon activity. Moreover, sustained IFN signaling can induce the expression of granulocyte colony-stimulating factor (G-CSF) through JAK-STAT activation, accelerating neutrophil differentiation and activation. This process promotes the upregulation of the NLRP3 inflammasome and the subsequent release of IL-1β, establishing a self-amplifying feedback loop that exacerbates synovial inflammation in osteoarthritis [166]. In psoriasis (PSO), high expression of IFN-λ1 in skin lesions fosters a pro-inflammatory environment that accelerates autoimmunity and tissue damage and appears to be driven by Th17 cells [167, 168]. Pathway enrichment analyses of upregulated genes in the lesional skin of patients with systemic sclerosis (SSc) have identified type I interferon, together with IRF and STAT families, as major upstream regulators of disease-associated transcriptional programs. Notably, the expression of IRF5, IRF7, IRF8, and IRF4 is strongly correlated with SSc pathogenesis [169]. Consistently, excessive expression of IFNs and IFN-induced chemokines has been observed in whole blood and peripheral blood mononuclear cells from SSc patients [170]. Among these, the chemokine CXCL4 has emerged as a key pathogenic amplifier, which can activate immune cells and promote type I interferon production through plasmacytoid dendritic cells (pDCs) [171]. When CXCL4 binds to specific receptors on immune cells (such as CXCR3), it induces the accumulation of cytoplasmic DNA, activating the cGAS-STING signaling cascade that amplifies type I interferon responses and drives B-cell production of autoantibodies, ultimately sustaining the interferon characteristic of SSc and perpetuating autoimmune inflammation [172, 173]. Collectively, these findings underscore the central role of the IFN network as a pathogenic amplifier that bridges innate and adaptive immune dysregulation in immune-related diseases, linking immune and non-immune cell compartments within a highly interconnected inflammatory circuit.

### Cancer

Acute induction of IFNs triggers robust and effective inflammation, whereas prolonged or dysregulated IFNs signaling can promote tumor progression. IFNs modulate cancer development through both direct effects on tumor cells and indirect regulation of the tumor microenvironment, including tumor-infiltrating lymphocytes and stromal components [174]. Sustained IFN-γ signaling, for instance, drives tumor cell adaptation by promoting STAT3-c-Myc-mediated metabolic reprogramming, shifting cellular energy metabolism from oxidative phosphorylation (OXPHOS) toward aerobic glycolysis [175, 176]. This metabolic switch not only facilitates tumor growth but also cooperates with the STAT1-PD-L1 axis to enhance immune evasion [31, 73]. Moreover, aberrant type I interferon signaling can contribute to metastasis and therapy resistance by promoting epithelial mesenchymal transition (EMT). This occurs through the upregulation of E-cadherin and activation of transcriptional programs that enhance cancer stem cell (CSC) properties, facilitating invasion, recurrence, and drug tolerance [11, 177]. In the context of brain metastasis, tumor cells exploit astrocyte-derived growth signals potentiated by IFN-α to activate NF-κB and STAT1 pathways, inducing a mesenchymal invasive phenotype and promoting metastatic colonization [178]. Similarly, in prostate cancer, IFN-α has been shown to activate dormant hematopoietic stem cells in vivo, potentially contributing to tumor relapse and systemic disease dissemination. In triple-negative breast cancer (TNBC), stabilization and persistent expression of IFNGR1 have been correlated with enhanced tumor growth, metastatic potential, and poorer clinical outcomes, underscoring the dual, context-dependent role of IFN signaling in oncogenesis [179]. On the other hand, persistent IFN signaling can facilitate tumor immune escape by suppressing the function of immune cells within the TME. Dysregulated signaling promotes the expression of inhibitory receptors, such as PD-L1, on tumor cells and tumor-associated macrophages (TAMs), and upregulates suppressor of cytokine signaling 2 (SOCS2) in DCs, contributing to TME reprogramming [180–183]. In addition, sustained interferon signaling leads to the dominance of selectively activated M2 macrophages and myeloid-derived suppressor cells (MDSCs), resulting in immunosuppression and invasion of tumor cells in an IFN-λ-and STAT3-dependent manner [90]. Chronic IFN-γ/JAK/STAT activation also upregulates tumor necrosis factor receptor superfamily member 14 (TNFRSF14) and galectin-9, which can reduce immune cell infiltration and foster long-term immune checkpoint blockade (ICB) resistance [76]. Takeda and colleagues demonstrated sustained IFN-γ signaling was shown to remodel tumor cell epigenetic responsiveness in murine models of breast cancer, lymphoma, and fibrosarcoma, conferring resistance to ICB [184, 185]. Moreover, sustained IFN-γ upregulates indoleamine 2,3-dioxygenase 1 (IDO1), which catabolizes tryptophan into kynurenine, a metabolite that suppresses T cell proliferation and function [186, 187]. In summary, the duration and intensity of IFN signaling represent two pivotal determinants of ISG expression patterns and epigenetic regulatory landscapes. These parameters critically define whether IFN exerts beneficial antitumor effects or detrimental pro-tumor consequences on both cancer and immune cells. The dynamic balance between these opposing outcomes ultimately dictates whether the immune equilibrium shifts toward effective immune control or, conversely, toward immune escape and tumor persistence.

### Neuroinflammation

A substantial body of evidence demonstrates that IFN signaling is closely interconnected with neuroinflammation within the central nervous system (CNS) and plays a pivotal role in the pathogenesis of various neurological and neurodegenerative disorders [188, 189]. Parkinson's disease (PD), a common age-related neurodegenerative disorder, is pathologically characterized by the selective loss of dopaminergic neurons in the substantia nigra pars compacta and the formation of Lewy bodies [190]. The elevated IFN-γ in cerebrospinal fluid and brain tissue of PD induces rapid upregulation of chemokines (CXCL9, CXCL10, CXCL11) in neurons and glial cell, promoting immune cell infiltration, neuroinflammation, and dopaminergic neuron loss, which is the pathological basis of motor dysfunction [191, 192]. Besides, Main et al. reported that knockout of the IFNAR effectively abolishes the 1-methyl-4-phenyl-1,2,3,6-tetrahydropyridine (MPTP)-induced type I interferon response, suppresses aberrant microglial activation and dopaminergic neuronal loss, and consequently mitigates neuroinflammation [193]. In Alzheimer’s disease (AD), IFN-induced transmembrane protein 3 (IFITM3) expressed in neurons and astrocytes regulates amyloid-β (Aβ) production and aggregation, aggravating neuroinflammation and neuronal loss [194–196]. Minter MR and colleagues also reported that IRF3 or IRF7 deficient cells exhibit greater resistance to Aβ-induced neurotoxicity compared with wild-type cells [197]. Accordingly, inhibition of interferon expression alleviates Aβ-driven neuronal death and cognitive decline in AD. TAR DNA-binding protein 43 (TDP-43), a predominantly nuclear DNA/RNA-binding protein, forms cytoplasmic aggregates that represent a hallmark pathological feature of amyotrophic lateral sclerosis (ALS). Yu et al*.* demonstrated that TDP-43 triggers the release of mitochondrial DNA into the cytoplasm, activating the cGAS-STING-IFN signaling axis and driving neuroinflammation in ALS [198, 199]. Moreover, mutations in the C9orf72 gene, recognized as the most common genetic cause of familial ALS and frontotemporal dementia (FTD), have also been linked to aberrant IFN signaling [200, 201]. McCauley et al. showed that STING blockade suppressed the hyperactive type I interferon response in C9orf72-deficient mice [202]. Moreover, in experimental autoimmune encephalomyelitis (EAE), mice with IFNLR deficiency showed reduced Th1 cell frequencies and cytokine production, while neutralization of type III interferon signaling improved recovery, indicating a pathogenic role for type III interferon in neuroinflammation [203]. Dysregulated type I interferon signaling has also been associated with inflammation-related depressive phenotypes, mediated by elevated TNF-α, IL-1β, and IL-6 levels that exacerbate neuronal injury and blood–brain barrier disruption [204–207]. In summary, dysregulated IFN signaling disrupts neurotransmitter homeostasis and accelerates the progression of various neuroinflammatory and neurodegenerative disorders through the excessive production of pleiotropic cytokines.

### Other diseases

With the deepening understanding of interferon biology, it has become evident that aberrant IFN signaling not only contributes to autoimmunity and tumor progression but also increases the risk of cardiovascular diseases [208]. Excessive activation of IFN pathways can drive endothelial dysfunction by inducing the expression of adhesion molecules and chemokines such as vascular cell adhesion molecule-1 (VCAM-1) and monocyte chemoattractant protein-1 (MCP-1) [209, 210]. Concurrently, IFN signaling suppresses endothelial nitric oxide synthase (NOS3) expression and nitric oxide production, enhancing proinflammatory leukocyte recruitment to the arterial wall and exacerbating the progression of atherosclerosis [211, 212]. In atherosclerotic models, IFN-β promotes leukocyte accumulation and plaque destabilization via CCL5-CCR5 signaling between endothelial cells and macrophages [213]. Prolonged IFN-α exposure damages endothelial cells, impairs vasodilation, disrupts endothelial progenitor cell (EPC) function, and accelerates thrombosis and platelet activation in murine lupus and non-lupus models [214, 215].

Myocardial infarction (MI) represents a pathological condition characterized by extensive cardiomyocyte death accompanied by acute inflammatory responses. Genetic deletion of either IRF3 or IFNAR has been shown to attenuate inflammatory activation and markedly improve survival in MI mouse models [216]. Similarly, hyperactivation of IFN pathways has been observed in patients with ulcerative colitis (UC), where colonic tissues from individuals with active disease exhibit significantly elevated expression of STING, TBK1, IRF3, and IFNB1 compared with healthy controls, supporting the activation of the STING-TBK1-IRF3 axis in UC pathogenesis [208]. Deficiency of the Atg16l1 gene in intestinal epithelial cells has been linked to spontaneous intestinal inflammation. In intestinal organoid models lacking epithelial Atg16l1, exogenous IFN-β markedly exacerbated cell death, whereas blocking IFN receptor signaling alleviated disease severity in vivo [217, 218]. These findings collectively indicate that suppressing excessive IFN activation may mitigate intestinal inflammatory immune responses and tissue injury.

Moreover, multiple clinical studies have demonstrated that individuals with autoimmune diseases characterized by heightened type I interferon signatures, such as SLE, are more susceptible to atherosclerosis and other cardiovascular diseases [219]. Proteomic profiling of platelets isolated from SLE patients revealed upregulation of IFN-regulated proteins, including IFITM1 and RPLKRA, which were strongly associated with previous cardiovascular events such as myocardial infarction and arterial or venous thrombosis. Together, these findings highlight a critical role for dysregulated interferon signaling in promoting both vascular inflammation and cardiometabolic pathology, suggesting that targeted modulation of the IFN axis may represent a promising strategy to mitigate inflammation-driven cardiovascular and gastrointestinal diseases.

Collectively, aberrant IFN signaling drives autoimmunity, tumor immune escape, neurodegeneration, and vascular inflammation. Gaining a deeper mechanistic understanding of the IFN-induced molecular circuits may open opportunities for the development of precision therapies that specifically attenuate pathological IFN signaling while preserving its protective functions.

## Therapeutic targeting of IFN pathways

### Interferon replacement therapy

The antiviral properties and immunomodulatory functions of interferons have generated significant interest in their clinical application for controlling viral infections, enhancing antigen presentation, and promoting antitumor responses. The therapeutic efficacy of IFNs is primarily attributed to three core mechanisms: antiviral activity, immune modulation, and inhibition of tumor cell growth. Different IFNs subtypes (e.g., IFN-α, IFN-β, and IFN-γ) exhibit distinct clinical applications, among which IFN-α remains the most widely used in practice. Recombinant IFN-α2b has been approved globally as adjuvant therapy for high-risk melanoma, and IFN-β remains standard of care in MS [220]. Viral vector-based strategies, such as adeno-associated virus (AAV)-mediated delivery, achieve sustained intratumoral type I interferon expression, selectively expanding antigen-specific T cells and driving tumor regression [221]. However, systemic IFN administration is limited by short half-life and tissue toxicity [220, 222]. PEGylation strategies have been employed to improve IFN pharmacokinetics by extending circulation time and reducing immunogenicity [223]. PEG-IFN-α2a has been shown to induce HBeAg seroconversion, a marker of sustained remission in chronic hepatitis B [224, 225], while PEG-IFN-α2b significantly improved relapse-free survival in high-risk melanoma patients [226]. A phase IIb clinical trial further demonstrated that pegylated IFN-λ enhanced NK and CD8⁺ T cell responses, leading to reduced HBV replication and antigenemia [227]. Beyond supplementation, therapeutic depletion of plasmacytoid dendritic cells (pDCs), the predominant source of type I interferon is being explored for autoimmune conditions such as SLE [228]. Importantly, timing and dosing remain critical determinants of efficacy, as excessive IFN signaling risks triggering autoimmunity or resistance.

### Small molecule drug development

Pharmacological inhibition of the JAK-STAT axis has emerged as a key strategy to mitigate pathological IFN signaling (Table 2) [234]. JAK inhibitors have demonstrated significant anti-inflammatory potential across diverse dermatological disorders. For example, ruxolitinib alleviating neuroinflammation and reducing HIV reservoir persistence, and baricitinib improving outcomes in inflammatory arthritis [234–236]. JTE-052 has been shown to suppress antigen-specific T cell activation and downstream inflammatory responses in models of contact hypersensitivity, irritant contact dermatitis, and other inflammatory skin diseases [237]. The monoclonal antibody VIB7734, directed against immunoglobulin-like Transcript 7 (ILT7), has shown efficacy in depleting circulating and tissue-resident pDCs in patients with cutaneous lupus erythematosus, attenuating local type I interferon activity and improving clinical outcomes [238, 239]. Pharmacological enhancement of autophagy may both fine-tune IFN activity and preserve T cell function [240, 241]. A study on intestinal inflammation shows that luteolin, a natural flavonoid, has demonstrated promising results by inhibiting the JAK/STAT pathway [242]. Taken together, these findings underscore the broad therapeutic relevance of small molecule inhibitors targeting the dysregulated IFN signaling pathway, extending from dermatological disorders to systemic autoimmune diseases. By targeting central nodes in cytokines signaling networks, IFN inhibitors offer a rational approach to rebalancing dysregulated immunity, and their integration into precision medicine strategies may further expand their clinical utility in inflammatory and immune-mediated diseases. Nevertheless, most small-molecule inhibitors remain in early development, and issues of specificity, pharmacokinetics, and off-target toxicity must be carefully addressed.

**Table 2:** Overview of inhibitors of IFN signaling pathway

Therapy	Mechanism of action	Effects	Phase	Disease focus	References
Ruxolitinib	JAK1/JAK2	Inhibits the infiltration of Th1 and Th17 cells	Approved by FDA (NCT03112603)	Psoriasis	[229]
Baricitinib	JAK1/JAK2	Improves disease activity	Approved by FDA (NCT01710358)	SLE\RA	[230]
Tofacitinib	JAK1/JAK3	Conserves the remission of ulcerative colitis	Phase II (NCT03288324)	RA\Psoriasis\SLE	[231]
Peficitinib	JAK1/JAK3	Ameliorates symptoms in desease	PhaseIII (NCT01565655)	RA	[232]
Decernotinib	JAK3	Improves signs and symptoms of RA	PhaseIII (NCT01830985)	RA	[233]

*SLE* Systemic lupus erythematosus, *RA* Rheumatoid arthritis

### Gene editing and CRISPR technology

The portability of CRISPR-Cas nucleases into eukaryotic cells has created vast opportunities for therapeutic gene editing to permanently correct a wide range of genetic disorders [243]. Heme oxygenase-1 (HO-1), a type II detoxifying enzyme, has been identified as a key modulator of radiation-induced type I interferon signaling [244, 245]. CRISPR-mediated HO-1 knockout disrupted the cGAS-STING pathway, enhancing STING-TBK1-IRF3-STAT1 phosphorylation, while paradoxically impairing downstream type I interferon production and reducing chemoresistance [246]. Similarly, loss of Vdac2 or Ptpn2 augments IFN-γ-driven JAK-STAT signaling, enhancing T cell cytotoxicity and antitumor immunity [247]. Parallel studies highlight loss of the histone methyltransferase Setdb1 in melanoma cells triggers intrinsic type I interferon signaling, upregulates MHC-I expression, and promotes CD8⁺ T cell infiltration, collectively improving immune clearance [248, 249]. Large-scale CRISPR screens have also uncovered regulators such as PTPN2 and APLNR, which modulate responsiveness to IFN-γ and influence immunotherapy efficacy [250, 251]. These findings underscore the utility of CRISPR in systematically mapping IFN signaling regulators, providing a foundation for next-generation immunotherapies through precision genome interrogation.

Together, these therapeutic avenues highlight IFN signaling as both a promising target and a delicate balance point in immunotherapy. While IFN replacement offers broad clinical benefit, small-molecule inhibition and CRISPR-based modulation provide unprecedented precision. Future breakthroughs will likely depend on integrating these strategies to contextually amplify beneficial IFN responses while constraining pathological signaling, thereby transforming IFN biology into a controllable therapeutic axis across infection, autoimmunity, and cancer.

## Future perspectives

### Precision medicine

**Fig. 6:**
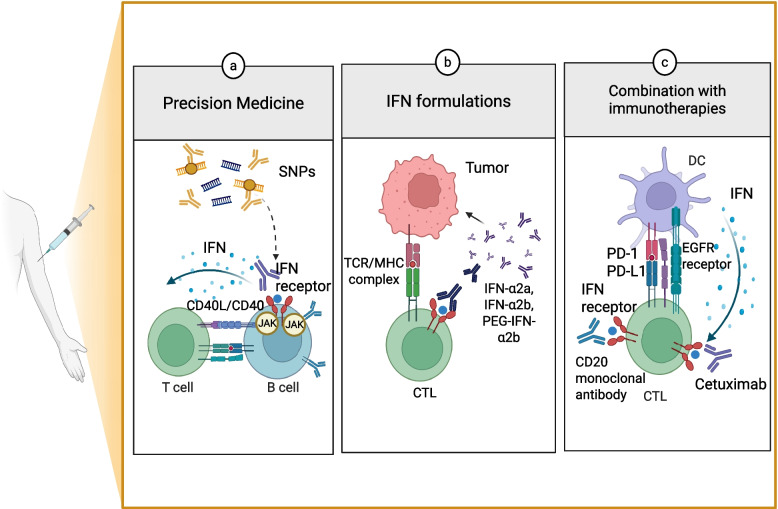
Therapeutic strategies targeting interferon (IFN) signaling in precision medicine and cancer immunotherapy. **a** Precision medicine: Genetic polymorphisms (SNPs) within IFN signaling components (e.g., IFN receptors, JAK kinases, transcriptional regulators) influence IFN responsiveness and therapeutic outcomes. These variations affect T-B cell crosstalk via CD40/CD40L and downstream JAK-mediated signaling, shaping adaptive immune responses. **b** IFN formulations: Recombinant and pegylated type I interferon (e.g., IFN-α2a, IFN-α2b, PEG-IFN-α2b) enhance antigen presentation via TCR/MHC class II interactions and promote cytotoxic T lymphocyte (CTL)-mediated tumor elimination. **c** Combination immunotherapies: Fusion of IFNs with monoclonal antibodies, such as IFN-α-anti-CD20 or IFN-β-cetuximab, augments antitumor immunity by engaging DCs, boosting CTL activation, and modulating PD-1/PD-L1 checkpoint pathways. Figure created with BioRender.com

In the era of precision medicine, IFN gene signatures have emerged as crucial biomarkers to enable personalized therapeutic strategies (Fig. 6). Numerous single nucleotide polymorphisms (SNPs) have been identified in genes associated with IFN signaling, including IFN receptor subunits, JAK kinases, and STAT transcription factor [252]. The functional impact of these SNPs depends on their genomic context, potentially altering transcriptional regulation, mRNA stability, alternative splicing, or protein activity [253]. When located within critical regulatory regions (e.g., ISREs, GAS, mRNA protective elements, etc.), these polymorphisms can significantly influence IFN gene expression, signaling efficiency, and therapeutic responsiveness [254]. SNPs in interferon regulatory factor 5 (IRF5) have shown the strongest clinical correlation with IFN-β responsiveness, serving as one of the most validated biomarkers for predicting therapeutic outcomes [255]. Functional IRF5 variants may influence IFN-β efficacy by altering the balance between Th1/Th17 versus Th2 helper T cell subsets, thus modifying the immune response dynamics [256]. As technological advances continue, the discovery of additional biomarkers linked to IFN responsiveness is anticipated. These biomarkers will further enhance our ability to predict individual treatment responses, guide rational therapy selection, and ultimately improve therapeutic outcomes and prognosis for patients undergoing IFN-based interventions.

### Combination therapies

IFNs were widely used either alone or in combination with chemotherapy to treat hematologic malignancies such as lymphoma, chronic myeloid leukemia, and hairy cell leukemia, as well as solid tumors including Kaposi sarcoma and renal cell carcinoma [257]. Type I interferon formulations (IFN-α2a, IFN-α2b, PEG-IFN-α2b) remain integral components of adjuvant therapy for high-risk melanoma following surgical resection [258, 259]. Targeting IFN signaling in combination with immunotherapies has emerged as a promising strategy to enhance treatment efficacy. For instance, fusing IFN-β with the epidermal growth factor receptor (EGFR)-targeted antibody cetuximab enables control of cetuximab-resistant tumors by inducing DC-mediated priming of CTLs [260]. Similarly, IFN-α fused with an anti-CD20 antibody demonstrates potent anticancer activity against CD20-expressing lymphoma and melanoma [261]. However, IFNs act as a double-edged sword. While they can amplify antitumor immunity, persistent IFN signaling contributes to resistance against immune checkpoint blockade (ICB) [262]. Combining IFN-based therapies with ICB (e.g., anti-PD-1 or anti-CTLA-4 antibodies) has shown potential in reversing T cell exhaustion and restoring durable antitumor immunity. In chronic HIV infection, sustained type I interferon activation promotes immune dysfunction and contributes to CAR-T cell exhaustion, posing a major barrier to therapeutic success [263]. Pharmacological modulation with rapamycin, an mTOR inhibitor, alleviates IFN driven dysfunction, restoring T cell metabolic fitness and enhancing CAR-T cytotoxicity against tumors [264]. Radiotherapy, which is administered to over half of advanced cancer patients, enhances immune priming via IFN induction but concurrently drives Serpinb9 expression, thereby inhibiting granzyme B and impairing CTL-mediated cytotoxicity [265–268]. Thus, Targeting Serpinb9 or its IFN-dependent regulatory axis may therefore enhance antitumor immunity and overcome radiation-associated resistance.

Advances in biomarkers, tailored IFN-based combinations, and integration with conventional therapies underscore the versatile therapeutic promise of IFN. However, their dual capacity to drive antitumor immunity or promote resistance remains a central challenge. Future efforts should dissect the temporal and tissue-specific dynamics of IFN signaling, refine predictive biomarkers, and optimize combinatorial regimens. Ultimately, precision-guided modulation may transform IFN into a cornerstone of personalized medicine across oncology, infectious disease, and autoimmunity.

## Conclusions and perspectives

With advances in mechanistic studies, the multifaceted roles of interferons have been firmly established beyond its classical antiviral core, centered on JAK-STAT-mediated induction of ISGs, to encompass roles in bacterial infection control, immune homeostasis, and modulation of inflammatory diseases. The differential tissue distribution of IFN receptor complexes, further underpins their site-specific immune functions. Notably, the same interferon may exert diametrically opposing effects depending on disease context, pathological stage, and host immune status, highlighting the necessity of evaluating IFN responses within their precise.

From a physiological perspective, IFNs act as central orchestrators of immune equilibrium, bridging innate and adaptive responses. By inducing antiviral effectors, activating DC and NK cells, and guiding T and B cell differentiation, IFNs establish a coordinated antiviral state essential for immune homeostasis. In cancer, IFNs enhance tumor immunogenicity through MHC upregulation, antigen presentation, and CTL recruitment, while triggering antiproliferative and pro-apoptotic programs that facilitate tumor elimination. However, chronic or dysregulated IFN signaling transforms this protective axis into a pathogenic one. Persistent activation drives autoantibody production and inflammation in autoimmune diseases such as SLE, PSO, and SSc, and exacerbates neuroinflammation and vascular injury in degenerative and metabolic disorders. Moreover, sustained IFN exposure paradoxically promotes tumor immune evasion by upregulating immunosuppressive molecules including PD-L1, IDO1, and SerpinB9, fostering a tolerant tumor microenvironment. Collectively, these observations underscore the context-dependent duality of interferons, as both guardians of host defense and potential mediators of immune pathology, highlighting the need for precise modulation of IFN signaling in therapeutic interventions.

Considering the dynamic and context-dependent functions of interferon signaling in cancer initiation, immune equilibrium, and metastatic progression, as well as its paradoxical impact on immunotherapy, it is imperative to design precision therapeutic strategies that align with disease-specific IFN signatures. Such context-guided interventions may balance efficacy with safety and overcome interferon-driven immune resistance. In contexts where interferon signaling is intrinsically compromised, such as in genetic immunodeficiencies, or actively suppressed by tumor-driven immune escape mechanisms, targeted augmentation of IFN pathways may reinvigorate immune surveillance and restore antiviral or antitumor immunity, ultimately improving disease outcomes. Exogenous IFN-α and IFN-β are used in clinical practice, including in viral hepatitis, multiple sclerosis, and certain hematologic malignancies. IFN fusions with monoclonal antibodies, such as IFN-β linked to the EGFR-targeted cetuximab or IFN-α fused to anti-CD20 antibodies, enhance tumor-specific immunity and overcome therapeutic resistance.

Conversely, during autoimmune disorders, severe infections, or cytokine storm syndromes, persistent interferon hyperactivation drives abnormal proliferation of immune cells and uncontrolled cytokines secretion, culminating in systemic inflammation and tissue pathology. Therapeutic attenuation of IFN signaling in these scenarios can restore immune balance, reduce inflammation, and protect against immune-mediated organ injury. Inhibitors of the JAK-STAT pathway, such as ruxolitinib and baricitinib, have demonstrated efficacy in autoimmune and autoinflammatory conditions by dampening chronic IFN signaling. More targeted approaches, such as monoclonal antibodies that deplete plasmacytoid dendritic cells (the major source of type I interferon), have entered clinical testing in lupus erythematosus. In radiotherapy, IFN induction enhances antigen release and immune priming but also drives upregulation of Serpinb9, thereby inhibiting CTL-mediated cytotoxicity. Targeting Serpinb9 or disrupting its regulatory axis may therefore unlock synergistic benefits of radiotherapy and IFN signaling.

Despite significant clinical successes, the therapeutic use of interferons still encounters substantial challenges. Systemic IFN therapy is frequently hampered by dose-limiting toxicities, such as influenza-like syndromes, hematologic suppression, neuropsychiatric disturbances, and autoimmune responses. In addition, multiple resistance mechanisms have been identified, including receptor downregulation, SOCS-dependent feedback inhibition, and epigenetic silencing of ISGs transcriptional programs, all of which compromise sustained therapeutic efficacy. To overcome these challenges, innovative delivery systems such as PEGylated interferons, viral vectors, nanoparticles, and cell type-specific targeting platforms are under active development. Moreover, precision medicine approaches are poised to reshape the landscape of interferon-based therapy. Interferon gene signatures and single-nucleotide polymorphisms (e.g., IRF5 variants) are emerging as predictive biomarkers to guide therapeutic decisions and prognosis. CRISPR-based genetic screens have identified novel regulators of interferon responsiveness (such as Setdb1 and PTPN2), revealing new therapeutic entry points. In addition, autophagy modulators and cGAS-STING inhibitors offer promising strategies to indirectly modulate interferon activity in chronic inflammation and cancer. The integration of multi-omics analyses with artificial intelligence is expected to accelerate the discovery of next-generation biomarkers and therapeutic targets.

Ultimately, IFNs serve as central orchestrators of host defense against infection and cancer, yet their signaling must be exquisitely tuned to maintain immune equilibrium. Aberrant interferon activity, whether through persistent overproduction or inadequate induction, can drive a spectrum of immune-mediated pathologies. Thus, the maintenance of a well-calibrated basal interferon tone is fundamental to immune homeostasis. Harnessing emerging technologies to dissect the context-dependent and subtype-specific functions of interferons will be crucial for designing next-generation therapeutic interventions that maximize clinical benefit while minimizing immune toxicity.

## Data Availability

Not applicable.

## Abbreviations

IFNs: Interferons
ISGs: Interferon-stimulated genes
ICB: Immune checkpoint blockade
JAKs: Janus kinases
PRRs: Pattern recognition receptors
PAMPs: Pathogen-associated molecular patterns
TAMs: Tumor-associated macrophages
ECM: Extracellular matrix
TME: Tumor microenvironment
MDSCs: Myeloid-derived suppressor cells
NO: Nitric oxide
CNS: Central nervous system
HO-1: Heme oxygenase-1
EGFR: Epidermal growth factor receptor
SNPs: Single nucleotide polymorphisms
IRF5: Interferon regulatory factor 5
ILT7: Immunoglobulin-like Transcript 7
VCAM-1: Vascular cell adhesion molecule-1
MCP-1: Monocyte chemoattractant protein-1
SLE: Systemic lupus erythematosus
